# Supplementary material for: Motivational factors were more important than perceived risk or optimism for compliance to infection control measures in the early stage of the COVID-19 pandemic
Source: PLoS One. 2022 Sep 23;17(9):e0274812. doi: 10.1371/journal.pone.0274812 (PMC9506657; doi:10.1371/journal.pone.0274812)
Supplement: Attachment — Submitted filename: Motivation, not optimism - response to peer review.docx [file pone.0274812.s001.docx]

# Response to peer review in PLOS One

Dear editor,

We are very grateful to the editor and the two anonymous reviewers for taking time to consider our manuscript. The reviewers made a number of comments and suggestions, all of which we found to be well-considered and meaningful. We have thoroughly revised all sections of the manuscript based on this review. How we have responded to each of the comments are detailed below, citing the revised manuscript text and their respective line numbers. Note that line numbers refer to the version of the manuscript where changes are not marked.

Since one of the comments suggested to adjust our sample, we have recalculated our analyses, and updated the numbers reported in the manuscript, as well as updating the files for dataset, analysis scripts, and analysis output on OSF. Note that adjusting sample did not substantially change the result of the analyses.

We hope that the editor and reviewers agree that these revisions have improved the clarity and the contribution of the manuscript.

Best wishes on behalf of both authors,

*Bjørn Sætrevik*

## Reviewer #1

### 1. The relationship between optimism and compliance to infection control measures has been investigated previously by at least two studies (Hartmann, M., & Müller, P. (2022). Acceptance and Adherence to COVID-19 preventive measures are shaped predominantly by conspiracy beliefs, mistrust in science and fear - A comparison of more than 20 psychological variables. Psychological Reports. [https://doi.org/10.1177/ 00332941211073656](https://doi.org/10.1177/%2000332941211073656); Jovančević, A., & Milićević, N. (2020). Optimism-pessimism, conspiracy theories and general trust as factors contributing to COVID-19 related behavior – A cross-cultural study. Personality and Individual Differences, 167, 110216. Error! Hyperlink reference not valid.). The results from these (and possibly other) previous studies should be integrated in the introduction and discussion of results.

#### Response:

We concede that our reference to previous literature may have been weak, especially when it comes to the relationship between optimism and compliance.

We have briefly introduced the Jovančević & Milićević paper on line 150: “*A study of convenience samples in Serbia and Latin-America from April 2020 [30] showed that optimism was associated with lower perceived risk and higher compliance.*” We also refer to this paper in the Discussion, on line 555: “*Similar to our study, previous studies have also shown optimism to have positive associations with compliance [e.g., 30].*”

We have also discussed the following article that addresses optimism and compliance: Reizer A, Munk Y, Frankfurter L K. (2022). Laughing all the way to the lockdown: On humor, optimism, and well-being during COVID-19. *Personality and Individual Difference,* 184: 111164. doi:10.1016/j.paid.2021.111164. This is presented on line 74: “*A recent study [18] showed that optimism was positively associated with well-being during the COVID-19 lockdown in Israel.*”

In addition, we have added a more direct account for the effect of optimism bias on compliance with reference to Weinstein (1987), see line 99: *“Having an optimistic bias when thinking about the pandemic Optimism bias could reduce compliance with infection control measures, as we think that we are relatively safe from infection, while this risk mainly threatens others. Thus, while dispositional optimism may have overall benefits in everyday life, it may be detrimental during a real threat such as a pandemic, as it lowers compliance [as shown for other health related advice, 23].”*

We have edited the manuscript to more explicitly discuss the complex relationship that may exist between optimism and compliance in the paragraph starting on line 105. Here have included a mention of the Hartmann & Müller paper that the reviewer suggests: *“* As suggested above, *optimism can have both positive and negative effects for health and well-being, and it may have particular effects for risk assessment and motivation factors during a specific threat such as a pandemic. The literature is not yet clear on this, and a recent study on a convenience sample in Switzerland [24] did not find an association between optimism and compliance. In the current manuscript we will explore these potentially complex relationships between optimism and compliance during the initial stage of a pandemic.”*

### 2. On p. 4, the authors argue that self-interested concern for your own well-being is a central motivation for compliance. This is certainly true, but what about the motivation to care for others, e.g., because many friends belong to the risk group, or if a person has a pronounced (pro)social behaviour? Also the big 5 trait agreeableness may play a role (Hartmann & Müller, 2022). Some of this is mentioned on p. 6, this could be integrated a bit better, also to avoid some redundancies in 1.1.2 and 1.1.3.

#### Response:

We agree that care for others may be a crucial motivation for compliance. This was indeed intended to be one of the main messages of the current paper. The section titled “Perceived pandemic risk” (line 49) was intended to show that self-interested concern has been given a main role in past research on pandemic compliance. But as the reviewer mentions, in the following sections we then go on to argue that prosocial motivation may be important for compliance (see line 136), and this is indeed one of our main findings that are discussed in particularly in the section “Motivation and compliance” (lines 601-630) of the Discussion.

We have revised our presentation of this issue, in particular in the Introduction, to avoid giving the impression that we think that perceived risk is in fact the crucial determinant for compliance in the current setting. We have made various small changes to the section starting on line 55, and have added the following sentence on line 68 to lead us over to the following section: *“However, variation in perceived risk may not be a crucial factor to explain variation in compliance in all pandemic settings, and other factors may be of equal or larger importance.”*

We have also more clearly stated this perspective in the Discussion, on line 529: “*It is thus possible that despite an optimistic bias making people see themselves as safe, them may nevertheless be motivated to comply in order to protect others that they see as being at risk,”* on line 614: *“It could be argued that people who mostly think of compliance in terms of protecting others (rather than protecting themselves) have more prosocial, rather than self-interested motivation for complying. In this sense our results indicate that prosocial motivation may be one of the crucial factors to explain variation in compliance to infection control measures”.*

We have tried to make this argument more clearly in a dedicated paragraph in the section “Optimism and compliance” in the Discussion (lines 547). *“However, our results indicated the inverse relationship, that optimism had a small positive association with following the infection control measures. This initially surprising finding indicates that a more optimistic mindset could contribute positively to compliance through providing motivation and drive to follow the measures [15]. It could be that pessimism, in the sense of not expecting the pandemic situation would improve over time or that oneself to manage its challenges, could decreased the motivation to comply with the measures. To comply with the measures involves changing and adapting our daily routines and activities, often to the detriment of quality of life. These costs of compliance combine with lacking certainty about how effective the measures will be effective in protecting oneself and others from a novel and unknown pandemic. Optimism may also be related to believing that the measures will be effective, which may drive the positive association between optimism and compliance. It should be noted that while past research tends to support an optimism bias, the assumed positive association between optimism and health protective behaviours has been more difficult to demonstrate [see e.g., 51].*”

### 3. Optimism is a complex issue. The authors mention that optimism is likely to be associated with lower risk perception and lower compliance to preventive measures. However, also the attitude of whether the measures or compliance will be effective determine actual compliance. The authors well addressed these different possible facets of optimism. For this study, I was wondering whether it would not have been of importance to differentiate optimism also for their measurements (e.g., general optimism that things come out well for you, optimism of dealing with infection, and optimism of the effect of compliance to the preventive measures. Why did the author not measure these different facets?

#### Response:

We agree that optimism is a complex issue, and that it could be of value to measure it as a general optimism trait, optimism towards a specific issue, and perhaps also e.g., optimism for being able to comply.

As can be seen in Table 1, we do have some items that approaches some of the issues that the reviewer is raising: We do not only ask about optimism in general (item 20), but also about optimism in how serious a potential infection would be (item 49), optimism in handling challenges related to the pandemic (item 50) and optimism about receiving effective treatment if sick (item 74). If we understand the reviewer correctly, they may be asking for more detailed reporting of these facets. Based on this we have now included more detailed reporting about how these single items relate to compliance. This can be found in the following text on line 379: *“The four “Optimism” items were scored on a scale from 1-5, and the average index approached high optimism (M = 3.78, SD = .49). As shown in Table 1, participants were very optimistic that they would receive good medical treatment if sick, and highly optimistic that they would handle the challenges of the pandemic. They were somewhat less optimistic in general, and were moderately optimistic about how serious it would be for them to be infected.”*

The reviewer also raises an interesting issue about whether the participants had “optimism about the effect of complying with the measures”. There is some discussion of similar issues, in particular on line 564 (*“Thus, an optimistic and positive mindset may have provided the necessary enthusiasm and perseverance to anticipate being able to comply over time.”*). We do have data to partially address this issue, based on responses to the item *“By following the infection control measures, I will avoid getting sick / making others sick”* (item 44b and 44c). We report the correlation between these items and compliance in the section “Prosocial motivation and compliance”, where we focus on the difference in effect between these two items. However, we could also use these items to indirectly indicate optimism in the measures’ effectiveness, as the reviewer is interested in. We have added a brief mention of this to line 553 of the Discussion: *“These costs of compliance combine with lacking certainty about how effective the measures will be effective in protecting oneself and others from a novel and unknown pandemic. Optimism may also be related to believing that the measures will be effective, which may drive the positive association between optimism and compliance.”*

### 4. 1.4.2 the hypothesis regarding information gathering is difficult to understand when the reader does not know how “information gathering” is operationalized. For example, “only trusting sources they find credible and trusting the official information” is ambiguous. Some may find sources of fake news and conspiracies very credible, then a reverse association with perceived risk may be expected. Please clarify at this point whether the measurement focuses on official information gathering.

#### Response:

We agree that the phrasing about “credible sources” in the materials is ambiguous, and this limits what conclusions we can draw from this measure. It is indeed possible that some participants think of conspiratorial sources as credible. In a previous publication (Sætrevik, 2022), the concept was discussed in more detail, including a discussion about the response distributions. We have now revised the text to have more precision in the way we refer to the concept of “information gathering”:

When introducing the concept on line 162 we have added the sentence: *“Such deliberation may take the form of being careful about which sources of information about the pandemic one considers to be credible, and to trust the information coming from those sources.”*

We now more clearly refer to the previous publication on line 204: *“Note that a previous publication presented more detailed item description, response distributions and visualization [41] from the same dataset. For brevity we will limit the descriptive reporting in the current paper, and instead encourage any reader interested in more detailed description of results to examine our previous paper.”*

When introducing the hypothesis on line 236 we now more clearly show how it relates to the operationalization: *“Individuals that see the pandemic as threatening are more likely to be more careful about how they gather information about the pandemic. This may have different effects on the information gathering. More careful information gathering may be expressed as trusting the official information about the pandemic. More careful information gathering may also lead to evaluating information sources in terms of their trustworthiness.”*

We now also make this clearer when returning to the issue in the discussion (line 638): *“Our descriptive results showed that a majority of the population emphasized both that information about the pandemic should come from credible sources and they trusted that information was not being kept away from them.“*

We have also noted the limitations of how the concept is measured on line 648: “*Note that people’s ideas of trustworthiness may differ, and what some consider a credible source may be considered as conspiratorial by official standards.”* We return to this issue under “Limitations” on line 682: *“As noted above, the operationalization of the concept of information gathering is somewhat open to interpretation as to what the participant considers to be a credible source.”*

### 5. p. 9 “the reported symptoms make it less likely that they had in fact been infected by the coronavirus”. This is a rather speculative statement. There are many infected people that “only” had such described symptoms without high fever. The author shuld at least report some references to support such a statement.

#### Response:

We agree with the reviewer’s comment, and have moderated this statement. In now reads (on line 255): *“Given that mild symptoms were difficult to discern from other respiratory infections at the time of data-collection, it is difficult to say whether the 122 participants that assumed they had been infected, had in fact been so.”* In our supplied script for preparing the dataset for analysis, we have now included frequency tables to show the frequency of symptoms for those who assume or have had confirmed infections.

### 6. Analysis: Given that the subsample of participants with confirmed infections was too low for separate analysis, they should probably be excluded from the analysis. Also, the people without confirmed infections might differ in terms of motivation. This should be captured by a covariate “infected”, or alternatively also this subsample could be removed from the analysis.

#### Response:

We assume that the first sentence of the comment is meant to refer to participants that have confirmed infections, whereas the second sentence is meant to refer to participants that assume but have not confirmed infections. We agree that it makes sense to exclude the 4 participants with confirmed infections. We also agree that it makes sense distinguish out the 122 participants who assumed they had been infected. It may be interesting to test how this sub-sample differs from the main sample on our research questions, like the reviewer suggested. However, this is somewhat outside the scope of our paper and we fear that including this may overly complicate the already extensive results reporting. For now, we therefore prefer to go for the other option the reviewer suggested, to exclude this sub-sample.

This reduces our dataset to N = 3.954. We have now implemented this in our analysis scripts, and have adjusted our results accordingly. This does not change any of the results significantly. We have added the output file of the analysis the reviewer is requesting to the online repository. We have added a sentence on line 255 explaining this: *“Given that mild symptoms were difficult to discern from other respiratory infections at the time of data-collection, it is difficult to say whether the 122 participant that assumed they had been infected by the coronavirus, had in fact been so. Only four participants stated to have had the coronavirus infection confirmed by a medical test. Since these participants may differ in their responses to other variables in the hypotheses, we have excluded them from the sample. As a result, hypotheses H4 and H5 related to effects of perceived risk and compliance on infections will therefore not be further discussed in the current paper. The provided data and analysis scripts should make it possible for interested readers to rerun the analyses with these participants reinstated.”*

### 7. I was surprised that the compliance measure included items about trusting the infection control measure. Such items rather capture attitude and not directly compliance in terms of actual adherence to measurements. The differentiation between attitude and behaviour was discussed in the introduction but seem to be ignored for the study.

#### Response:

Thanks for evaluating the materials so carefully. It is true that of the four items we use to measure compliance, item 45 asks about “trusting the health authorities’s advice about the pandemic”, and items 44b and 44c asks about the motivation for compliance, while item 44a directly adresses the intention to comply. To make this clearer, we have added the following sentence when introducing the measure on line 358: *“One item asked about trusting the advice about the infection control measures, two about being motivated for following the measures, and one about intending to follow the measures.”*

If we understand the reviewer correctly, they raise the issue that including items related to attitudes and motivation in addition to intention to comply can make the concept overly broad and ambiguous. However, it could also be argued that attitudes, motivation and intentions are all related to the actual act of complying (which we after all cannot measure in a survey study). It could therefore me seen as valuable to include all the aspects in the measure. Furthermore, our preregistration included all these four items as part of our compliance measure, as it should thus be reported correspondingly in the manuscript.

Inspired by this issue being raised by the reviewer we have added a supplemental analysis where all the hypothesis tests that involving compliance are tested against the singe item about intention to comply (excluding the items about attention and motivation). This has been described on line 464 of the revised manuscript: *“Note that our measure of compliance included questions about attitudes and motivation in addition to intention to comply. In order to test the effect on intention to comply alone, we also ran the tests of H1d and H2a on the single item for compliance, instead of the index across all compliance items. The effect of H1d remained significant but of a weaker effect. H2a showed a significant effect in the predicted direction with a negligible effect size. These analyses can be found as supplemental online materials (*[*https://osf.io/khbvz/*](https://osf.io/khbvz/)*).”*

### 8. p. 15 “most trusted the pandemic information” -> the “official” information is meant here, right?

#### Response:

Thanks for spotting this. We have made the relevant change under the section “Response distributions” on line 393 to: *“…most trusted the official pandemic information…”*

### 9. Did the author test for possible covariates for the regression analyses? For example, compliance might be higher for older people (or particularly for people in the risk group). Was there any health-related information about the participants as to whether they belong to the risk group (preconditions)? These people possibly have a higher compliance that may be independent from optimism etc. Or do you think this is sufficiently captured by higher perceived risk? Maybe this issue should be discussed somewhere more explicitly.

#### Response:

Some of the demographic variables such as age do indeed have an impact on outcome variables like compliance as the reviewer is asking about. These are reported on in a previously published (open access) article. We have rewritten this section to make it clearer what information is available in the earlier publication (on line 301): *“A previous publication [41] from the same dataset provides more details about the data collection, and provides descriptive statistics for the sample, figures showing the response distributions, and discusses some covariates.”*

The reviewer raises an interesting point about health conditions as a covariate for the compliance and optimism regressions. Unfortunately, we do not have a good measure of the underlying health conditions of the participants. Also, as the reviewer mentions, the possible impact of preconditions may already be captured by the “perceived risk” variable. Especially considering the item asking about whether they believe that they will be seriously ill if infected with COVID-19. Thus, adding “risk group” or “medical vulnerability” as a covariate may be redundant.

### 10. In addition to reporting F-statistics, the authors could provide a summary graph of their hypothesis where the relevant regression estimates and their 95%Cis are depicted.

#### Response:

Note that Reviewer #2 also made a similar comment about visualizing the results as their first issue. Please see our response there.

## Reviewer #2

**The authors report data collected during the early stages of the COVID-19 pandemic from a large Norwegian sample. Overall, I find the study well conducted. In particular, hypotheses were pre-registered and results are clearly reported and carefully discussed in the manuscript. To make it brief, I am very much in favor of seeing this manuscript published. I only have three comments:**

### 1. Please show figures depicting the main regressions (or correlations). I would be very much interested in seeing the distributions of the measured variables. In some cases, there might be floor or ceiling effects (or heavily skewed distributions) that would affect the interpretation.

#### Response:

Thanks for this suggestion. We have added figures 1, 2 and 3 to highlight what we think of as the most important regressions. Note that Reviewer #1 also made a similar comment #10 about visualizing the results.

### 2. What are the relationships between the questions in one category, i.e., between the items grouped into the five variables in Table 1? Cronbach’s alpha would be a standard measure. For some items, the authors discuss specific effects but it would be generally important to see if the categorization of these items is warranted (based on the obtained empirical data).

#### Response:

We have included inter-item reliability measures for each of the variable categories. Note that these were listed under the “Variables” section under “Materials and Methods”, rather than under “Results”, which may have made them difficult to locate. We would be happy to move them, if that would make more sense. We report Cronbach’s alpha where appropriate, coefficient omega or Spearman’s r where they are more suitable. Note that some of these measures indicate low cohesion, but we find this acceptable as they should be considered reflective measures, rather than formative measures of a latent variable.

### 3. The text is very clearly written with one exception. The term optimism is used in different circumstances. Sometimes it is used as a short form for “optimism regarding the risks of COVID-19.” Sometimes it is used as a more general measure. It is also a bit confusing in some cases how “optimism” is different from “optimism bias.” The authors could simply provide a table that lists the different usages of these terms and then stick to these throughout the manuscript (including the title and the abstract).

#### Response:

Both reviewers commented on how we use and define the term “optimism”. This may have been somewhat confusing in the previous version of the manuscript, since we could have been clearer in the way we introduced the term, and since we refer to slightly different aspects of favorable expectations throughout the manuscript. We use “optimism” in three ways in the manuscript, and we have tried to improve clarity in the introduction section of the manuscript by better distinguishing between trait optimism (i.e., dispositional optimism, first paragraph starting on line 71), from optimism about being able to handle pandemic challenges (second paragraph, starting on line 82), and from the influence of optimism bias in evaluating one’s own risk compared to the risk for others (fourth paragraph, starting on line 92).

Reviewer #2 also suggests a table that lists the different usages of these terms. We believe that this function is to some extent already served by using different subheadings for each terms in the section “Background” (from line 56 and onwards), where each paragraph starts with a definition of the term. We have tried to further structure this section to define the concepts more clearly in the first sentence. Further, we believe the operationalizations provided in Table 1 provides reference point for each of the concepts. As far as we can see, tables are not frequently used in PLOS One to introduce concepts.
